# Supplementary material for: The causal role of circulating inflammatory markers in osteoporosis: a bidirectional Mendelian randomized study
Source: Front Immunol. 2024 Jul 18;15:1412298. doi: 10.3389/fimmu.2024.1412298 (PMC11291241; doi:10.3389/fimmu.2024.1412298)
Supplement: Supplementary file 1 [file DataSheet_1.zip › supplementary material/CIM-OSTEOPOROSIS /GCST90274821.csv_leave_one_out.pdf]

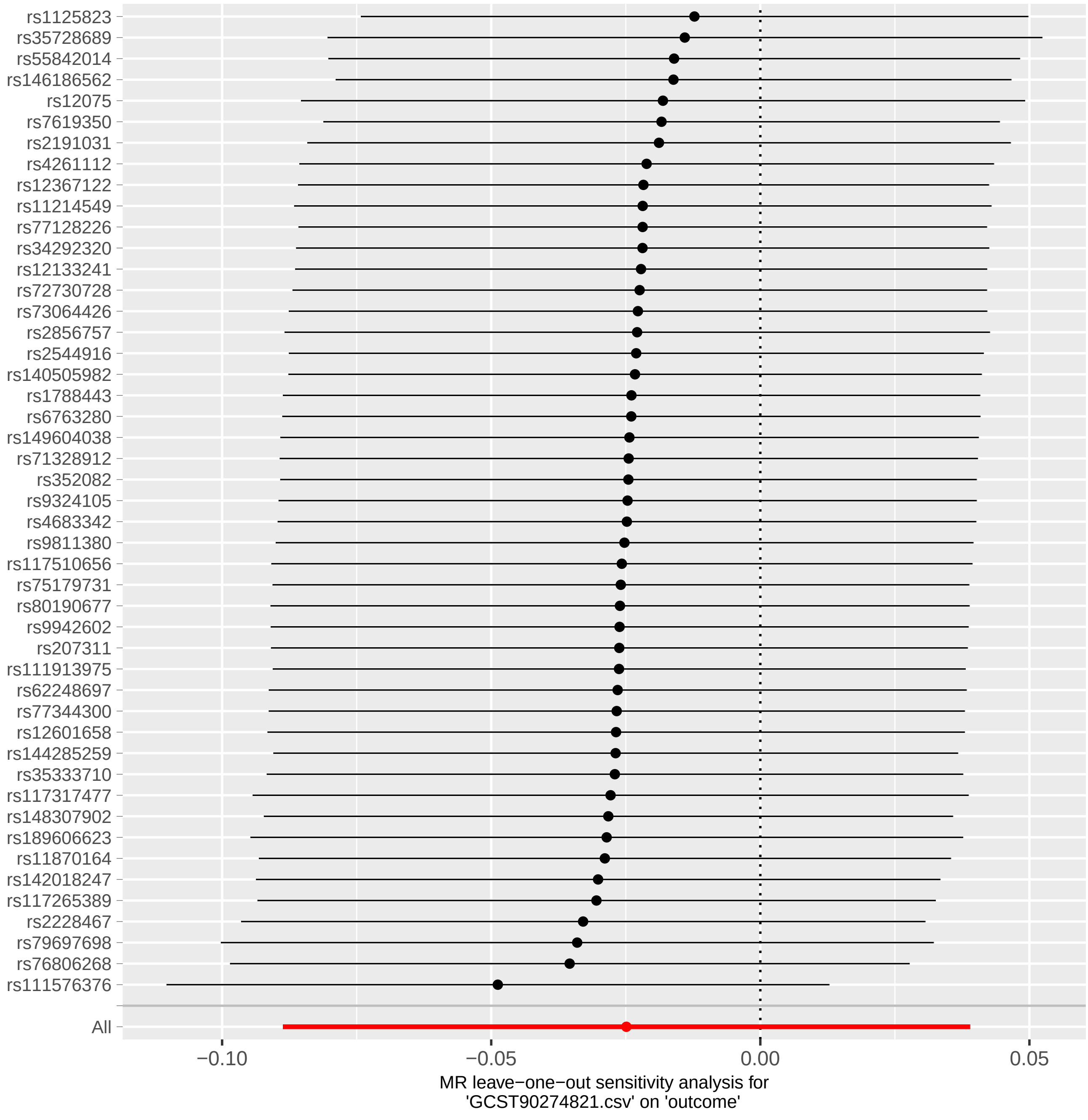
rs1125823
rs35728689
rs55842014
rs146186562
rs12075
rs7619350
rs2191031
rs4261112
rs12367122
rs11214549
rs77128226
rs34292320
rs12133241
rs72730728
rs73064426
rs2856757
rs2544916
rs140505982
rs1788443
rs6763280
rs149604038
rs71328912
rs352082
rs9324105
rs4683342
rs9811380
rs117510656
rs75179731
rs80190677
rs9942602
rs207311
rs111913975
rs62248697
rs77344300
rs12601658
rs144285259
rs35333710
rs117317477
rs148307902
rs189606623
rs11870164
rs142018247
rs117265389
rs2228467
rs79697698
rs76806268
rs111576376
All
−0.10
−0.05
0.00
0.05
MR leave−one−out sensitivity analysis for
'GCST90274821.csv' on 'outcome'
